# Multiple trauma is linked with reversal of immunoparalysis and provides survival benefit from Pseudomonas aeruginosa

**DOI:** 10.1186/cc13427

**Published:** 2014-03-17

**Authors:** E Mandragos, A Pistiki, DI Droggiti, M Georgitsi, E Giamarellos-Bourboulis

**Affiliations:** 1 Attikon Hospital, Chaidari, Attiki, Greece; 2 University of Athens, Medical School, Athens, Greece

## Introduction

Patients with multiple injuries are prone to hospital- acquired infections. We hypothesized that exposure to multiple injuries may modulate the innate immune response and the subsequent outcome of infections.

## Methods

Ninety-seven C56Bl6 male mice were subject to multitrauma after crush of the femur and chemical pneumothorax by turpentine. Mice surviving 72 hours after the injuries were challenged intravenously with one 7log_10 _log-phase inoculum of P. aeruginosa and survival was recorded. In separate experiments, mice were sacrificed post injury; splenocytes were isolated and stimulated with 10 ng/ ml LPS and cytokines were measured in supernatants by an enzyme immunoassay. Quantitative cultures of the right lung, kidney and liver were performed. The same procedures were done for sham-operated mice and for mice subject only to femur crush and to pneumothorax.

## Results

**Figure 1:**
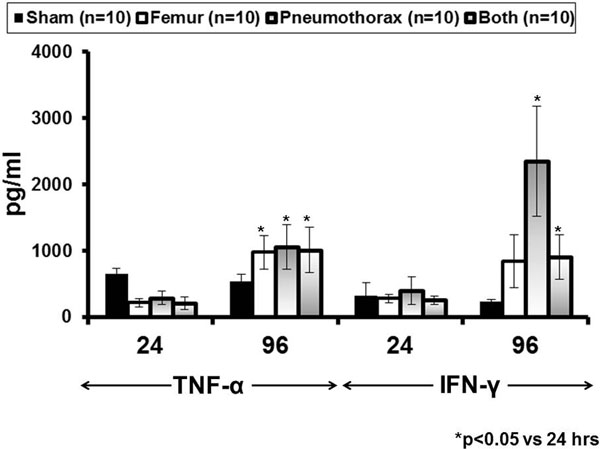
**Cytokine stimulations after sterile injury**.

Initial experiments with 21 mice showed that the overall death rate for this model of multitrauma was 66.7% with most deaths occurring in the first 48 hours. In the second set of experiments, 12 mice remaining alive 72 hours post injury were challenged with *P. aeruginosa*; mortality was 37.5% compared with 75% of 12 noninjured and infected mice (log-rank: 5.77, *P *= 0.016). Respective mean production of TNFα from splenocytes isolated at sacrifice 24 hours and 96 hours post sham injury was 651 and 523 pg/ml (*P *= NS); post femur crush 217 and 916 pg/ml (*P *= 0.010); post pneumothorax 286 and 1,056 pg/ml (*P *= 0.018); and post both femur crush and pneumothorax 207 and 1,011 pg/ml (*P *= 0.019). Respective mean production of IFNy from splenocytes isolated at sacrifice 24 hours and 96 hours post sham injury was 324 and 230 pg/ml (*P *= NS); post femur crush 278 and 840 pg/ml (*P *= 0.021); post pneumothorax 377 and 2,356 pg/ml (*P *= 0.025); and post both femur crush and pneumothorax 252 and 908 pg/ml (*P *= 0.019). Mean production of TNFα from splenocytes of injured mice sacrificed 24 hours after bacterial challenge was 1,184 pg/ ml compared with 484 pg/ml non-injured and infected mice (*P *= 0.024). Similar differences were not found for IFNy or for quantitative tissue cultures. See Figure 1

## Conclusion

Mice survivors from multiple trauma become resistant to subsequent infection. This is probably linked with the induction of tolerance of the innate immunity to substances released after sterile tissue injury.

